# Cyanidiales-Based Bioremediation of Heavy Metals

**DOI:** 10.3390/biotech12020029

**Published:** 2023-04-18

**Authors:** Hari Lal Kharel, Ina Shrestha, Melissa Tan, Mohammad Nikookar, Negar Saraei, Thinesh Selvaratnam

**Affiliations:** 1 Department of Civil and Environmental Engineering, Lamar University, Beaumont, TX 77705, USA; hkharel@lamar.edu (H.L.K.); ishrestha@lamar.edu (I.S.); mtan2@lamar.edu (M.T.); mnikookar@lamar.edu (M.N.);; 2 Center for Advances in Water & Air Quality, College of Engineering, Lamar University, Beaumont, TX 77705, USA

**Keywords:** biosorption, Cyanidiales, *Galdieria sulphuraria*, heavy metals, removal efficiency

## Abstract

With growing urbanization and ongoing development activities, the consumption of heavy metals has been increasing globally. Although heavy metals are vital for the survival of living beings, they can become hazardous when they surpass the permissible limit. The effect of heavy metals varies from normal to acute depending on the individual, so it is necessary to treat the heavy metals before releasing them into the environment. Various conventional treatment technologies have been used based on physical, chemical, and biological methods. However, due to technical and economic constraints and poor sustainability towards the environment, the use of these technologies has been limited. Microalgal-based heavy metal removal has been explored for the past few decades and has been seen as an effective, environment-friendly, and inexpensive method compared to conventional treatment technology. Cyanidiales that belong to red algae have the potential for remediation of heavy metals as they can withstand and tolerate extreme stresses of heat, acid salts, and heavy metals. Cyanidiales are the only photosynthetic organisms that can survive and thrive in acidic mine drainage, where heavy metal contamination is often prevalent. This review focuses on the algal species belonging to three genera of Cyanidiales: *Cyanidioschyzon*, *Cyanidium*, and *Galdieria.* Papers published after 2015 were considered in order to examine these species’ efficiency in heavy metal removal. The result is summarized as maximum removal efficiency at the optimum experimental conditions and based on the parameters affecting the metal ion removal efficiency. This study finds that pH, initial metal concentration, initial algal biomass concentration, algal strains, and growth temperature are the major parameters that affect the heavy metal removal efficiency of Cyanidiales.

## 1. Introduction

Heavy metals (HMs) are released into the environment and can become hazardous when disposed of without proper treatment, as they can accumulate in living organisms. The most common HMs are arsenic (As), cadmium (Cd), chromium (Cr), copper (Cu), iron (Fe), lead (Pb), mercury (Hg), nickel (Ni), and zinc (Zn) [1,2]. These HMs are released into the environment through anthropogenic activities, such as mining and smelting, industrial production, domestic use of metals, and agricultural utilization [3,4]. These HMs enter water bodies through industrial discharges, domestic sewage, nonpoint runoff, urban storm runoff, and atmospheric precipitation [5]. HM pollution in the water bodies occurs when water cannot self-purify due to high concentrations of HMs. In order to safeguard surface water and groundwater from HMs, various environmental laws and regulations have been implemented [6].

The release of HMs into the environment has threatened plants, animals, humans, and the ecosystem. Most HMs become toxic when exposed to environmental factors such as soil, air, water, and other living organisms due to their bioaccumulating, teratogenic, mutagenic, and non-biodegradable characteristics [4]. These HMs can enter the human body in a variety of ways and can result in health issues and symptoms ranging from mild to severe, such as headaches, arthralgia, mental disorders, altered liver and kidney function, or even cancer [7]. When HMs accumulate in the human body, they deplete essential nutrients and negatively interact with the cells, decreasing immunological defenses [8]. Some of the negative effects of HMs on humans are summarized in Table 1.

There are many conventional techniques for removing HM ions from aqueous solutions based on physical, chemical, and biological methods, including adsorption, ion exchange, chemical precipitation, and electrochemical treatment [13,14]. Due to technical and economic constraints and poor sustainability, the use of traditional techniques has been limited [6]. Due to these limitations of the traditional treatment technique, phycoremediation has emerged as a viable alternative in HM remediation. Over the past few decades, phycoremediation has been explored as a more sustainable, effective, environment-friendly, and inexpensive method compared to other conventional physiochemical techniques. In the phycoremediation process, microalgae are considered to remove the impurities from water and wastewater using microalgae’s ecological functions [15]. Many studies have been conducted based on biosorption and bioaccumulation processes to develop more effective ways of removing HMs.

The increased use of microalgae for HM removal is due to exceptional biological features, such as having the ability to grow well in harsh environmental conditions, high photosynthetic efficiencies, high binding affinity, various binding sites, and large surface area [9]. Many researchers have explored the use of different types of microalgae (green, red, and brown) as potential biosorbents for HMs. Cyanidiales are unicellular red algae that have the potential to be used for the remediation of HMs as they can survive in extreme environmental conditions such as high temperatures (37–55 °C) and very low pH (0.5–3.0) [16]. They can also withstand and tolerate extreme stresses of acid salts and HMs [16,17]. Compared to other microalgal species, Cyanidiales are unique in their ability to adjust to extreme environmental conditions, including high temperatures and low pH, as well as its resistance to high salt and numerous toxic metals [18].

Despite the numerous review articles available on phytoremediation-based HMs removal, the use of living Cyanidiales in HM removal has not been fully explored yet [12,13,15,19,20,21,22]. Considering this, this review attempts to compile the published research activities focused on using algal species belonging to three genera of Cyanidiales: *Cyanidioschyzon*, *Cyanidium*, and *Galdieria* for the application of HM removal.

This review paper covers the use of Cyanidiales for HM bioremediation documented in the literature from 2015 to date. Extensive literature searches were conducted mainly using the Web of Science and the terms “Cyanidiales” and “Heavy metals” were initially used to scan the titles. Additional keyword searches were also conducted using the terms “Red algae”, “bioremediation”, and “phycoremediation”. The references of the papers that were acquired were also searched for additional relevant articles. A total of 11 records were ultimately included for data extraction, all of which are specifically focused on the study of the use of living Cyanidiales in HM bioremediation. The quality of the paper was evaluated based on the number of times a particular article was referenced in other works. Additional research papers were gathered to create a summary for HM removal using the conventional treatment process and the treatment process based on the green algae.

## 2. Conventional Treatment Technologies for Heavy Metal Removal

HMs cannot degrade chemically or biologically in the environment and hence enter the environment through various means resulting in HM pollution. Therefore, treating toxic, non-biodegradable, and persistent HM is paramount before directly discharging them. Some of the most readily available conventional methods include chemical precipitation, membrane filtration, coagulation and flocculation, electrodialysis, flotation, ion exchange, solvent extraction, and adsorption.

Adsorption is the adhesion of extremely thin molecules layers at the solids’ surface by physical forces or chemical bonds [8]. It is a process that involves the mass transfer between the solid and liquid phases. Initially, the pollutant penetrates the adsorbent surface resulting in the adsorption of the pollutant on the surface, and then complete sorption of the pollutant takes place after penetration in the adsorbent structure [23]. Previously, activated carbon was mostly used as a standard adsorbent. However, due to its high cost, several natural substances, such as kaolin, rice husk, seaweed, etc., are being used as adsorbents nowadays for HM uptake from aqueous solution [24].

Chemical precipitation is a technique where a chemical reaction of a metal ion with a precipitating agent transforms the dissolved metal ions into insoluble solid phases [25]. The addition of precipitating agents to a solution containing metal ions will cause metal ions to react with the hydroxide ions and form an insoluble metal hydroxide precipitate, where the precipitate is separated either by sedimentation or filtration [23].

Ion exchange is an electrostatic interaction that exchanges cations and anions. It is a reversible process where an insoluble material (resin) takes ions from an electrolytic solution and releases other ions in a chemically equivalent amount by maintaining the resin’s structural integrity [26]. Synthetic resins with sulfonic acid groups, carboxylic acid groups, and natural zeolites are used to exchange their cations with the metals [3].

Membrane filtration technique is an effective and promising option for the removal of HMs because of their high efficiency and convenience. Membrane filtration is a physical separation method for removing certain substances from a solution by applying high pressure to feed water driven through a semipermeable membrane [8]. Membrane filtration processes are classified as reverse osmosis, ultrafiltration, nanofiltration, and electrodialysis according to their pore size, membrane permeability, and operating pressures [23].

Electrochemical techniques enable the recovery of metals in their elemental metal state by plating out metal ions on a cathode surface. Electrocoagulation (EC), electro floatation (EF), and electrodeposition are three well-established technologies based on electrochemical methods, with EC being the most prevalent [3]. EC is the in situ production of coagulants by dissolving aluminum or iron ions from aluminum or iron electrodes using an electrical current. The anode is responsible for producing metal ions, whereas the cathode is responsible for releasing hydrogen gas. Hydrogen gas aids in removing flocculated particles from water by causing them to rise to the surface [3,27].

For several decades, physical and chemical advances were extensively used to remove and recover HMs with varying degrees of success. Some of the initiatives and technological developments achieved so far in conventional treatment are summarized in Table 2.

However, these conventional technologies have several disadvantages, such as intensive energy requirements, incomplete removal of metal ions, secondary toxic sludge production, and high operation and maintenance costs [9,12,22]. Realizing the need for more effective, inexpensive, and environment-friendly technologies, microalgal-based HM removal techniques have become popular.

## 3. Algal-Based Heavy Metal Removal

Biomaterials such as algae, plants, bacteria, fungi, yeasts, and biological wastes have been widely used as adsorbents for metal removal [17]. When compared with microbial organisms such as yeast and different fungi, algae prove to be the most efficient adsorbent for HM removal [6]. Algae are excellent bio-remediators because of their high tolerance capacity, high binding affinity, large surface area, and eco-friendly and reusable features [40]. Algal-based treatment is more convenient because algae can be used for many biotechnological applications, such as carbon reduction, biofuel generation, and bioremediation of impurities [1].

Biosorption is the primary mechanism in HM uptake by either active or passive algal biomass. Biosorption is a two-step process in which the initial step involves the adsorption of metal ions onto the cell surface. This process is relatively fast and may or may not involve metabolic activities. However, in the final step, the metal ions transport in the internal route of living cells and finally accumulate in the cytoplasm. This process is relatively slow and requires metabolic activities [5,22].

The presence of functional groups primarily influences metal biosorption. The algae cell wall consists of various functional groups such as hydroxyl, carboxyl, amide, thiols, phosphate, and hydroxide [20]. The functional groups are responsible for metal ion binding as they can produce negative charges on the surface of algae and cause an anionic effect on the cell wall [41]. However, functional groups in all the biosorbents are unlikely to guarantee the successful biosorption of pollutants [11]. Metal biosorption is also influenced by the structure and functionality of extracellular polymeric substances (EPS) produced by microalgae. EPS improves adsorption capacity, reduces intracellular accumulation, and increases the tolerance against metal ions by forming the extracellular protective layer on the surface of the cell wall [15].

Based on the above-mentioned factors, each algal strain’s performance can vary, influencing the sorption capacity and removal efficiency of the algal strain for HM bioremediation. Among the different types of algae (red, green, and brown) green algae are mostly used for bioremediation and have comparatively good removal efficiency. Table 3 summarizes the HM removal efficiency of a few green algal species published in the literature.

## 4. Cyanidiales and Their Effectiveness in Heavy Metal Removal

Cyanidiales are a type of red microalgae (RMA) which belongs to the Rhodophyta division and Cyanidiophyceae class. There are three genera and seven species of Cyanidiales. The three genera are *Cyanidioschyzon*, *Cyanidium*, and *Galdieria*, while the seven species include *Cyanidioschyzon merolae*, *Cyanidium caldarium*, *Galdieria sulphuraria*, *Galdieria partita*, *Galdieria maxima*, *Galdieria daedala*, and *Galdieria phlegrea* [17,49]. The Cyanidiales are the only photosynthetic organisms that can survive in extreme acidic and hot environments like hot sulfur springs. They inhabit the springs as biofilms and establish endolithic populations in the nearby rocks [50].

The three genera of Cyanidiales differ in their cellular size and shape. The genus *Cyanidioschyzon* has a smaller and oval shape cell and lack of cell wall, whereas the other two genera, *Cyanidium* and *Galdieria*, have similar morphological features with a cell of spherical shape and surrounded by a thick cell wall [51]. Until 1981, the genus *Cyanidium* was thought to be a synonym for *Galdieria* because of the similar morphological characteristics [52].

Each Cyanidiales utilizes a different substrate for its growth and can grow in different environments. *Galdieria sulphuraria* can grow in autotrophic, mixotrophic, and heterotrophic conditions by using a variety of organic substances as a carbon source. In contrast, *Cyanidioschyzon merolae* and *Cyanidium caldarium* can only grow in autotrophic growth mode [53,54]. *Cyanidium caldarium* and *Cyanidioschyzon merole* utilize nitrate and ammonium as nitrogen sources, whereas the growth of *Galdieria sulphuraria* is more prominent in ammonium and organic substrates [55].

The low pH and high-temperature need of Cyanidiales make them a potential biotechnological candidate because they reduce the likelihood of widespread culture contamination [56]. Extremely acidic environments promote metal solubility, creating an environment where metal-tolerant Cyanidiales can thrive [57]. A wide range of application possibilities is provided by Cyanidiales, including purifying contaminated soils and waters and exploiting their mixotrophic lifestyles to facilitate the effective production of bioproducts like phycocyanin and floridosides [50]. Among the three Cyanidilaes species, *Galdieria sulphuraria* is found to be well-suited for practical applications in biotechnology because of its advantageous mass production capabilities and lack of biological contamination [58,59,60,61,62,63].

### 4.1. Efficiency of Cyanidiales for Heavy Metal Removal

Different researchers have explored the ability of Cyanidiales to remove HM ions from an aqueous solution by considering different operational parameters. These parameters include growth medium, pH, temperature, light intensities, algal strains, initial biomass concentration (IBC), and metal concentration (IMC). The results of the most recent studies on HM removal using Cyanidiales at optimum experimental conditions have been tabulated in Table 4.

Reviewing the papers published after 2015, *Galdieria sulphuraria* was found to be the most frequently used species among different species of Cyanidiales. *Galdieria sulphuraria* was used by Minoda et al. [64] for the recovery of rare earth elements: neodymium (Nd), dysprosium (Dy), lanthanum (La), and Cu. The results showed that the removal efficiency of the Nd, Dy, La, and Cu ions was over 90% at a metal concentration of 0.5 mg L^−1^ and experimental pH of 2.5. Similarly, *Galdieria sulphuraria* was also used by Ju et al. [66] for metal recovery, which showed a significant recovery of Au, Pd, and Pt from the metal ion solutions. Above 95% of Au, Pd, and Pt were removed from the solution at a pH level of 2.5 and an IMC of 0.5. Compared to the results, the removal effectiveness of Au was higher than that of Pd and Pt. Fukuda et al. [54] also evaluated the metal accumulation ability of *Galdieria sulphuraria* for cesium (Cs). The best results were obtained in an optimum experimental condition where 52% of Cs was recovered.

In a study completed by Iovinella et al. [68], the removal of four rare earth elements (REEs): yttrium (Y), cerium (Ce), europium (Eu), and terbium (Tb) was achieved using two strains of *Galdieria sulphuraria.* SAG 107.79 and ACUF 427 were two strains subjected to metal solutions at pH levels ranging from 2.5 to 5.5. The results showed that all the removal abilities depended on the strain and pH. The maximum sorption capacity was observed in strain SAG 107.79 at pH 5.5, whereas ACUF 427 had a maximum sorption capacity (SC) at pH 2.5. A new strain of *Galdieria sulphuraria*, SBU-SH1 KY651246, was used by Jalali et al. [41] to find the biosorption efficiency for the three metals uranium (U), vanadium (V), and titanium (Ti). The study was conducted for 10 mg L^−1^ to 30 mg L^−1^ of the IMC by using 24 mg L^−1^ of *Galdieria sulphuraria* biomass within 48 h of contact time. The maximum SC of U was 333.23 mg g^−1^ at the IMC of 20 mg L^−1^, the maximum SC of Ti was 397.29 mg g^−1^ at the IMC of 30 mg L^−1^, and the maximum SC of V was 371.86 mg g^−1^ at the IMC of 25 mg L^−1^.

Ostroumov et al. [67] conducted experiments using *Galdieria sulphuraria* to remove four toxic HMs: Cd, Cu, Pb, and Ni. After an incubation time of 30 days, Ni showed the least amount of reduction in concentration at 19% among the four metals. The average concentration reduction for Cd was around 24%, and the average reduction in Pb levels was much greater at 84%. Moreover, Cu showed the greatest reduction in concentration at 95%. The removal efficacy of HM is also impacted by the method of biomass analysis. In another set of experiments carried out by Ostroumov et al. [65], the two methods used for the removal of Pb and Cu were stripping voltammetry and vitrified mortmass. While using stripping voltammetry, the biosorption of Cu was observed, while the biosorption of Pb was not. On the other hand, the vitrified mortmass of the same type of algal biomass showed no Pb or Cu sorption.

Three species of Cyanidiales, *Cyanidium caldarium*, *Cyanidioschyzon merolae*, and *Galdieria maxima* were used by Cho et al. [17] to determine their capacities for Pb biosorption. From the results, *Cyanidium caldarium* showed the highest capacity for Pb sorption with 298.4 mg g^−1^, whereas *Galdieria maxima* had the lowest Pb sorption capacity with 38.2 mg g^−1^ at pH level 5.0. Regarding *Cyanidioschyzon merolae*, the sorption capacity at pH 5.0 was 214.0 mg g^−1^. Likewise, Cho et al. [49] used *Cyanidium caldarium*, *Cyanidioschyzon merolae*, and *Galdieria partita* to determine their capacities for Cr (VI) sorption. Among the studied Cyanidiales, *Cyanidioschyzon merolae* showed a higher capacity for Cr (VI) sorption with 168.1 mg g^−1^, whereas the sorption capacity of *Cyanidium caldarium* and *Galdieria partita* was 151.7 mg g^−1^ and 103.9 mg g^−1^ at pH level 2.0.

The REEs present in the luminophores, e-waste, were sorbed using another Cyanidiales species, *Galdieria phlegrea*, in a study conducted by Čížková et al. [69]. The result showed that Y was the most biosorbed metal ion in *Galdieria phlegrea*, with a sorption capacity of 11.14 mg g^−1^. The sorption capacity for Ce, La, and Eu was 0.23, 0.57, and 0.90 mg g^−1^, respectively. The observed results may be because Y positively affects photosynthesis and stimulates the chlorophyll synthesis of *Galdieria phlelgra.*

While Cyanidiales have been used for HM bioremediation, the mechanisms behind this phenomenon are not well explored. Living algae employ various defense mechanisms to lessen the HM’s impact, as previously discussed. The possible defense mechanisms involve chelation, complexation, biosorption on the functional group, ion exchange, intracellular localization in the organelles (e.g., vacuole, chloroplasts, and mitochondria), and enzymatic and biochemical transformation [15]. These defense strategies for preventing the toxic effects of HM offer an excellent opportunity for biotechnological purposes [16]. Cyanidiales are ideal for bioremediation because they have either a genetic or physiological adaptation that enables them to withstand numerous metals. It is hypothesized that biofilm development and metal detoxification are both aided by the production of EPS in Cyanidiales [57]. The other mechanisms involved in the Cyanidiales based studied are biosorption [64], bioleaching and biosorption [41], complexation with organic functional groups and transport to vacuole [17], biosorption and formation of polysaccharides complexes [49].

### 4.2. Effects of pH on Heavy Metal Removal by Cyanidiales

pH is one factor affecting the removal efficiency of metal ions from the solution. pH level affects the functional group present on the surface of the algae. Generally, at lower pH, the protonation of functional groups occurs which induces conflict between H^+^ ion and HMs for binding with the functional group. The efficiency of the protonated functional group is less compared to the normal functional group. On the other hand, as the pH rises, the total negative charge on the surface of the algae will increase because of the deprotonation of a functional group; hence, HMs are more likely to be adsorbed on the surface of the algae through various interactions [20]. The results of the most recent studies using living Cyanidiales at different pH conditions have been tabulated in Table 5. The experiments were performed by altering the pH at constant IMC and IBC.

The effect of pH on metal recovery was considered by Minoda et al. [64]. The results showed that, in the case of Nd, Dy, and La, the recovery rate reached a maximum at pH level 2.5 and decreased between 35% to 55% when pH increased from 2.5 to 5.0. At pH levels between 1.0 and 2.5, the recovery rate was more than 65%. In contrast, the recovery rates for the Cu ions considerably decreased at pH levels below 2.0 while they were found to be stable at pH levels between 2.0 and 5.0.

To study the effect of pH, an experiment was carried out by Ju et al. [66] at a minimum IMC of 0.5 mg L^−1^ and a maximum IBC of 14 g L^−1^. When the pH level drops from 2.5 to 0.5, there is a minimal decrease in removal efficiency in Au and Pd while there is a significant decrease in removal efficiency (95% to 10%) in the case of Pt.

The effect of pH was studied by Iovinella et al. [68], for the removal of REEs: Y, Ce, Eu, and Te using two strains of *Galdieria sulphuraria:* SAG 107.79 and ACUF 427. The range of pH levels was 2.5 to 5.5. The results showed that removal abilities were dependent on pH. In both strains, a significant increase in metal removal was observed when the pH level increased from 2.5 to 5.5.

Cho et al. [49] used *Cyanidium caldarium*, *Cyanidioschyzon merolae*, and *Galdieria partita* to remove the Cr (VI) at two different pH levels: 2.0 and 7.0. In all the strains, when the pH level was increased from 2.0 to 7.0 the sorption capacity decreased from 151.7 to 87.7 mg g^−1^ in *Cyanidium caldarium*, from 168.1 to 73.0 mg g^−1^ in *Cyanidioschyzon merolae*, and from 103.9 to 93.7 mg g^−1^ in *Galdieria partita.*

### 4.3. Effects of Initial Metal Concentration on Heavy Metal Removal by Cyanidiales

IMC is another factor that also influences removal efficiency. At higher IMC, the filling of active sites on the surface of the algae will occur, resulting in lower biosorption of HMs. In addition, at lower IMC there is more possibility of diffusion of HMs through the algae surface, therefore maximizing the removal efficiency [70]. The results of the most recent studies using Cyanidiales at different IMC conditions have been tabulated in Table 6. The experiments were performed by altering the IMC at constant pH and IBC.

In a study conducted by Minoda et al. [64], it was found that the concentration of metal ions influenced the recovery rate. The metal recovery experiment was conducted at IMC ranging from 0.5 to 25 mg L^−1^. At a metal concentration of 0.5 mg L^−1^, the removal efficiency was more than 95% and decreased to around 50% when the concentration rose to 25 mg L^−1^ in the case of all studied metals (Nd, Dy, La, and Cu). Likewise, Ju et al. [66] also studied the effects of IMC and found that when IMC gradually increased from 0.5 to 25 mg L^−1^, the removal efficiency of Au and Pd did not decrease significantly, while in the case of Pd, it gradually decreased from 95% to 70% at algal biomass density of 14 g L^−1^ and from 85% to 20% at algal biomass density of 1.4 g L^−1^.

### 4.4. Effects of Other Factors on Heavy Metal Removal by Cyanidiales

Apart from pH and IMC, other factors such as temperature, growth mode, and IBC also affect the HM removal efficiency. However, very few studies have been conducted for the above-mentioned parameters. For the effect of growth mode, Minoda et al. [64] conducted a study using five different development environments: autotrophic, mixotrophic, heterotrophic, semi-anaerobic autotrophic, and semi-anaerobic heterotrophic. Out of five different development environments, effective recoveries were obtained from a solution in a semi-anaerobic heterotrophic environment. Another research experiment was conducted by Fukuda et al. [54] where *Galdieria sulphuraria* was grown in autotrophic, heterotrophic, and mixotrophic growth modes. The best results were obtained in a mixotrophic condition.

Minoda et al. [64] also attempted to study the effect of temperature. The temperature is known to affect the biological activity of algae. The ideal temperature for algal growth is typically around 40 °C, so the biological activity was low at 4 °C. The recovery rates of the metal ions decreased between 30–40% at 4 °C in living cells. Considering how the dying cells showed no metal recovery at 40 °C or 4 °C, the findings indicate that recovery of metal ions in a semi-anaerobic heterotrophic environment is the biological mechanism. To study the effect of IBC, Ju et al. [66] performed a study at an IBC of 1.4 g L^−1^ and 14 gL^−1^ of biomass for the removal of Au, Pd, and Pt. In the case of Au and Pd, the removal efficiency is almost half at 1.4 g L^−1^ compared to using 14 g L^−1^. However, in the case of Pt, no removal efficiency was observed when using the algal cell of 1.4 g L^−1^, while efficiency reached around 80% when using the cells of 14 g L^−1^.

### 4.5. Tolerance of Cyanidiales for Heavy Metal Toxicity

The selection of metal-tolerant microalgae is the key step when the phycoremediation process is based on living cells. Living microalgae produce a variety of EPS in metal-stressed conditions, and the ability of microalgae to clean up metallic pollution is increased by the role that EPS can play in the sorption of metal ions [71]. However, very few studies have been conducted on the tolerance of HM toxicity by an algal strain.

Sirakov et al. [16] used four Cyanidiales species: *Galdieria sulphuraria*, *Galdieria maxima*, *Galdieria phlegrea*, and *Cyanidium caldarium* to investigate the tolerance capacities and growth abilities of these species in an aqueous solution containing Au and Pd ions. The growth of Cyanidiales was observed at different metal concentrations ranging from 1 g L^−1^ to 10 g L^−1^. Regarding *Galdieria sulphuraria*, Au inhibited cell proliferation at 1 g L^−1^ and 10 g L^−1^ compared to the control. However, in the case of Pd, there was a slower growth rate at 1 g L^−1^ as compared to 10 g L^−1^, suggesting that this algal strain grows more in higher Pd concentrations. For *Galdieria maxima*, the presence of Au severely inhibited cellular growth at all concentrations examined, while the presence of Pd had no detrimental effects on cell growth. Both metal ions caused a tendency of reduced growth rate in *Galdieria phlegrea* at all metal concentrations tested. In the case of *Cyanidium caldarium*, Pd was tolerable to this species at all concentrations while Au greatly reduced the ability of cells to grow as the metal concentration rose.

From the above-mentioned discussion, among the species of Cyanidiales, *Galdieria sulphuraria* was found to be frequently used in HM removal. The removal efficiency varies on the targeted metals, the type of algae used, and the combination of metals. The experimental setup conditions such as pH, temperature, IBC, and IMC significantly affect the removal efficiency. Different strains of algae and the sources of HMs also influence the removal efficacy of the metals. If all the experimental conditions are met, almost 100% removal efficiency can be achieved using *Galdieria sulphuraria* for the removal of HMs.

### 4.6. Comparison of Removal Efficiency of Cyanidiales with Other Class of Algae

Based on the above research, it can be concluded that the effectiveness of HM removal varies with experimental factors, algal species, and the metals being removed. Unless all conditions are identical, it is challenging to compare the removal efficiency of Cyanidiales with that of other algal species. Although more studies have been conducted using green algal species, it is still unclear whether they are more effective at removing pollutants than other algal types. Table 7 summarizes the effectiveness of Cyanidiales and a few other algal species in removing particular HM. The results of this analysis show that the removal efficiency of Cu (II) is greater when using Cyanidiales species. In comparison, the removal efficiency of Cd (II) and Pb (II) is greater when using other algal species.

## 5. Challenges, Limitations, and Future Perspectives of the Phycoremediation Approach

Although microalgae can be used to remediate HMs, they still face several challenges for widespread use, including contamination by other microorganisms, nutrient variability, high levels of turbidity and total suspended solids, harvesting microalgal biomass, and downstream processing difficulties [9]. Further challenges also lie in the algal biomass’s small particle size, low chemical resistance, and low mechanical strength [12]. Harvesting is one of the most expensive processes in microalgae-based treatment techniques as biomass needs to be harvested regularly to produce on a large scale. In addition, harvesting techniques need to be developed in such a manner that dead biomass can be effectively removed from the growing biomass [72].

The need for a huge quantity of microalgae for large-scale industrial applications is another challenge in microalgal-based treatment techniques since the current yield of these techniques is relatively small. In light of this evidence, an effort has been made to estimate the mass of algal biomass required for Cu removal from a Superfund site managed by the Environmental Protection Agency. From 1860 through 1963, the Iron Mountain Mine in Shasta County, California, was mined for Au, Cu, silver (Ag), pyrite (FeS_2_), and Zn. All mining operations ceased in 1963, and the mine was put on the Superfund list in 1983. During the cleanup, from 2008 to 2012, two billion gallons of acid mine drainage (AMD) were treated, preventing the release of about 870,000 pounds of copper into the environment. At this Superfund site, 220 kg of Cu on average per day have been taken out from the AMD [73]. Using the yield value obtained from Minoda et al. [64] (1.38 mg Cu g^−1^ of algal biomass), around 159 metric tons of algal biomass are required daily at this specific Superfund site to bioremediate the Cu from the AMD.

Given the numerous benefits but few challenges that microalgae offer for HM bioremediation, future research should concentrate on scaling up microalgae’s ability to biosorb. Further investigation into upstream cultivation and downstream purification is necessary to achieve the large-scale bioremediation of HM as a cost-effective technology. Considering difficulties with the industrial implementation of the biosorption process and incomplete removal of pollutants, the development of hybrid technologies for the removal of HMs is also suggested. In addition, the underlying biosorption and bioaccumulation mechanisms of HM by microalgae must also be understood to create complete equilibrium and kinetic models for scaling up applications [7].

While using Cyanidiales for large-scale bioremediation of HM is not new, the related application has surprisingly seen no published data. All the experiments described in the reviewed papers considered were carried out on a laboratory scale. This may be because scalability in large-scale development faces significant obstacles, including the need to account for environmental variables like temperature and light when cultivating algae. Most of the related studies are still in the early stages with respect to technology readiness levels.

There is very little research on the sustainability assessment in regards to risk, environmental, and human health in HM removal by microalgae. Conducting a life-cycle analysis to determine the risk involved is strongly advised, particularly in the upscaling phase. The three thematic areas of risk assessment, hazards, and mitigation techniques have gaps in the literature that need to be filled by future studies [2].

Another major gap found in the study is performing all the experiments in synthetic HM solutions. Synthetic solutions may not properly reflect the characteristics of contaminated wastewater. In real-world situations, HMs are frequently found with other contaminants and organic matter. Scholars could overlook HMs’ interactions and interferences with other environmental compounds using synthetic solutions. This is also a major area that needs to be addressed in future research.

## 6. Conclusions

Effluents containing HMs have been treated using a variety of traditional technologies during the past few decades, including adsorption, ion exchange, chemical precipitation, and electrocoagulation. Phycoremediation, on the other hand, is an effective and inexpensive technology that can overcome the constraints of more traditional approaches. Cyanidiales, a type of red microalgae, is exceptional in adapting to a wide range of environmental stresses, including high heat, low pH, high salt, and several toxic metals. This review focuses on the Cyanidiales and their potential for HM removal under various experimental conditions and the influence of various parameters that change the efficiency of Cyanidiales. *Galdieria sulphuraria* is the most frequently used species among the Cyanidiales and can remove almost 100% of HM from the aqueous solution at optimum experimental conditions. pH, IMC, IBC, algal strains, and temperature primarily influence the HM bioremediation efficiency. However, most studies still lack essential information, such as suitability for large-scale application, performance in real wastewater effluents, environmental impacts, estimations of energy requirements, as well as capital investment and operational cost of the system. Taking these knowledge gaps into account, this review provides a foundation for future studies on HM bioremediation by using Cyanidiales.

## Figures and Tables

**Table 1:** Health effects of heavy metals.

Heavy Metals	Effects on Human	References
As	Chronic poisoning, cancer in different organs (lung, skin, kidney, and bladder), arsenical dermatitis, cardiovascular disease, diabetes, infant morbidity, hepatic damage, and hyperkeratosis.	[7,9]
Cd	Hyperglycemia, high blood pressure, peripheral neuropathy, osteoporosis, Parkinson’s and Alzheimer’s disease, damage to reproductive organs, kidney, liver and lungs, gastrointestinal disorder, amyotrophic lateral sclerosis, hypertension, renal dysfunction, bone degeneration.	[7,9,10,11]
Cu	Major cell damage, interruption in the food chain, growth and developmental abnormalities, carcinogenesis, neuromuscular control defects, mental retardation, anemia, gastrointestinal diseases.	[10,12]
Cr	Skin inflammation, liver damage, ulcers, chronic bronchitis, epigastric pain, tissue neurosis, internal hemorrhage, emphysema, DNA impairment.	[7,9]
Hg	Damage to the heart, brain and other organs, mental retardation, reproductive disturbance, Parkinson’s disease, Alzheimer disease, autism.	[7,9,11]
Ni	Gastrointestinal distress, pulmonary fibrosis, skin dermatitis, lung and kidney problems.	[3]
Pb	Impaired voluntary muscle function, mental retardation, encephalopathy, anemia, dementia, kidney malfunction and physical impairments, epilepsy, organ failure, coma, and even death.	[7,9,11]
Zn	Dizziness, nausea, major cell damage, interruption in the food chain, growth abnormalities, carcinogenesis, neuromuscular control defects, mental retardation.	[10,12]

**Table 2:** Conventional treatment processes for heavy metal removal.

Treatment Type	Targeted Metals	Initial Metal Concentration (mg L^−1^)	Removal Efficiency (%)	References
Adsorption	Cd (II)	3.22	83.38	[28]
	Pb (II)	4.17	99.90	
	Cd (II)	100.00	68.50	[24]
	Cu (II)		99.10	
	Pb (II)		99.80	
	As (V)	1000.00	53.00	[29]
	Cr (VI)	100.00	89.00	
	Pb (II)	1036.00	100.00	[30]
Chemical Precipitation	Mn (II)	1085.00	99.30	[31]
	Cd (II)	150.00	99.70	
	Zn (II)	450.00	99.80	
	Cu (II)	100.00	99.37–99.69	[25]
	Cr (III)			
	Pb (II)			
	Zn (II)			
	Cr (III)	5363.00	>99.00	[32]
Electrochemical Treatment	Pb (II)	9.00	96.70	[33]
	Zn (II)	3.20	95.20	
	Cr (IV)	1470.00	100.00	[34]
	Cu (II)	250.00	>96.00	[27]
	Ni (II)			
	Zn (II)			
Ion Exchange	Ni (II)	39.22	74.80	[35]
	Pb (II)	1036.00	55.00	[30]
	Zn (II)	327.00	100.00	[36]
Membrane Filtration	Cu (II)	500.00	99.50	[37]
	Ni (II)			
	Cr (IV)	60.00	96.50	[38]
	Ni (II)	133.00	99.20	
	Cd (II)	200.00	95.00	[39]
	Pb (II)		93.00	

**Table 3:** Summary of green algal species and their respective heavy metal removal capacity.

Algal Strain	Targeted Metals	Initial Metal Concentration (mg L^−1^)	pH	Sorption Capacity (mg g^−1^)	Removal Efficiency (%)	References
*Chlamydomonas reinhardtii*	Cu (II)	0.03	6.0	0.056	28.00	[5]
	Pb (II)	0.10		0.057	8.00	
*Chlorella* *vulgaris*	Cd (II)	100.00	NA	16.34	95.20	[6]
*Chlorella* *vulgaris*	Cd (II)	2.50	7.2	49.00	72.00	[42]
*Chlorella* *vulgaris*	Cu (II)	0.45–1.65	6.2	NA	92.53	[43]
	Pb (II)	1.95–4.83			98.70	
*Coelastrella* sp.	Cd (II)	2.50	7.2	65.00	82.00	[42]
*Chlorella minutissima* UTEX 2341	Cd (II)	67.48	6.0	35.36	74.34	[44]
	Cu (II)	63.55	4.0	3.28	83.60	
	Mn (II)	329.64	6.0	21.19	83.68	
	Zn (II)	392.28	6.0	33.71	62.05	
*Desmodesmus* sp. MAS1	Cu (II)	0.50	3.5		40.00	[45]
	Fe (III)	10.00			80.00	
	Mn (II)	10.00			40.00	
	Zn (II)	10.00			70.00	
*Oedogonium westti*	Cd (II)	0.50–2.00	5.0	0.974	95.00	[46]
	Cr (III)	0.50–2.00		0.620	93.00	
	Ni (II)	0.50–2.00		0.418	89.00	
	Pb (II)	0.10–0.80		0.261	96.00	
*Scenedesmus almeriensis*	As (II)	12.00	9.5	5.50	40.70	[47]
	B (III)	60.00	5.5	16.00	38.60	
*Ulva lactuca*	As (II)	1.00	7.8	0.30	48	[48]
	Cd (II)	0.20		0.07	56	
	Cr (III)	2.00		0.53	72	
	Cu (II)	1.00		0.37	86	
	Hg (II)	0.05		0.03	98	
	Mn (II)	2.00		1.06	74	
	Ni (II)	2.00		0.73	77	
	Pb (II)	1.00		0.44	87	

**Table 4:** Summary of Cyanidiales for heavy metal removal at optimum experimental conditions.

Algal Strain	Targeted Metals	Initial Metal Concentration (mg L^−1^)	pH	Sorption Capacity (mg g^−1^)	Removal Efficiency (%)	References
*Galdieria sulphuraria*	Nd (III)	0.50–25.00	0.5–5.0		35.00–100.00	[64]
	Dy (III)					
	La (III)					
	Cu (II)					
*Galdieria sulphuraria*	Cu (II)	1.90	2.4		100.00	[65]
	Pb (II)	0.10			0.00	
*Galdieria sulphuraria*	Au (III)	0.50–25.00	0.5–2.5		10.00–100.00	[66]
	Pd (II)					
	Pt (IV)					
*Galdieria sulphuraria*	Cs	0.30	2.5		52.00	[54]
*Galdieria sulphuraria* IPPAS P-513	Cd (II)	5.00	2.7		24.00	[67]
	Pb (II)				84.00	
	Ni (II)				19.00	
	Cu (II)				95.00	
*Galdieria sulphuraria* SBU-SH1 KY651246	Ti (IV)	10.00–30.00	2.5	397.29		[41]
	V (III)			371.86		
	U (VI)			333.23		
*Galdieria sulphuraria* SAG 107.79	Ce (III)	24.90	2.5–5.5	0.35–4.61		[68]
	Eu (III)	27.00		0.37–6.53		
	Tb (III)	28.30		0.39–5.74		
	Y (III)	15.70		0.22–2.78		
*Galdieria sulphuraria* (ACUF 427)	Ce (III)	24.90	2.5–5.5	2.94–5.97		[68]
	Eu (III)	27.00		3.56–6.52		
	Tb (III)	28.30		3.54–6.44		
	Y (III)	15.70		2.00–2.52		
*Galdieria phlegrea*	Ce (III)	3.80	4.0	0.23		[69]
	Eu (III)	17.31		0.90		
	La (III)	4.80		0.57		
	Y (III)	272.88		11.14		
*Galdieria maxima*	Pb (II)	500.00	5.0	38.20		[17]
*Galdieria partita*	Cr (VI)	500.00	2.0–7.0	93.70–103.90		[49]
*Cyanidium caldarium*	Pb (II)	500.00	5.0	298.80		[17]
*Cyanidium caldarium*	Cr (VI)	500.00	2.0–7.0	87.70–151.70		[49]
*Cyanidioschyzon merolae*	Pb	500.00	5.0	214.00		[17]
*Cyanidioschyzon merolae*	Cr (VI)	500.00	2.0–7.0	73.00–168.10		[49]

**Table 5:** Summary of Cyanidiales for heavy metals removal at different pH conditions.

Algal Strain	Targeted Metals	pH	Sorption Capacity (mg g^−1^)	Removal Efficiency (%)	References
*Galdieria sulphuraria*	Nd (III)	2.5		100.00	[64]
	Dy (III)			100.00	
	La (III)			98.00	
	Cu (II)			95.00	
	Nd (III)	5.0		50.00	
	Dy (III)			35.00	
	La (III)			55.00	
	Cu (II)			100.00	
*Galdieria sulphuraria*	Au (III)	2.5		100.00	[66]
	Pd (II)			95.00	
	Pt (IV)			95.00	
	Au (III)	0.5		95.00	
	Pd (II)			85.00	
	Pt (IV)			10.00	
*Galdieria sulphuraria* SAG 107.79	Ce (III)	5.5	4.61		[68]
	Eu (III)		6.53		
	Tb (III)		5.74		
	Y (III)		2.78		
	Ce (III)	2.5	0.35		
	Eu (III)		0.37		
	Tb (III)		0.39		
	Y (III)		0.22		
*Galdieria sulphuraria* (ACUF 427)	Ce (III)	5.5	5.97		[68]
	Eu (III)		6.52		
	Tb (III)		5.44		
	Y (III)		2.24		
	Ce (III)	2.5	2.94		
	Eu (III)		3.56		
	Tb (III)		3.54		
	Y (III)		2.00		
*Galdieria partita*	Cr (VI)	2.0	103.90		[49]
		7.0	93.70		
*Cyanidium caldarium*	Cr (VI)	2.0	151.70		[49]
		7.0	87.70		
*Cyanidioschyzon merolae*	Cr (VI)	2.0	168.10		[49]
		7.0	73.00		

**Table 6:** Summary of Cyanidiales for heavy metals removal at different initial metal concentrations.

Algal Strain	Targeted Metal	Initial Metal Concentration (mg L^−1^)	Removal Efficiency (%)	References
*Galdieria sulphuraria*	Nd (III)	0.50	100.00	[64]
	Dy (III)		100.00	
	La (III)		98.00	
	Cu (II)		95.00	
	Nd (III)	25.00	50.00	
	Dy (III)		50.00	
	La (III)		45.00	
	Cu (II)		60.00	
*Galdieria sulphuraria*	Au (III)	0.50	100.00	[66]
	Pd (II)		95.00	
	Pt (IV)		95.00	
	Au (III)	25.00	100.00	
	Pd (II)		95.00	
	Pt (III)		70.00	

**Table 7:** Use of Cyanidiales and other algal species for the removal of Cu (II), Cd (II), Pb (II).

Targeted Metals	Algal Strain	Class	Initial Metal Concentration (mg L^−1^)	pH	Removal Efficiency (%)	References
Cu (II)	*Galdieria sulphuraria*	Cyanidiophyceae	0.50	2.5	95.00	[64]
			5.00	2.7	95.00	[67]
	*Chlorella* *vulgaris*	Trebouxiophyceae	1.60	6.2	92.53	[43]
	*Ulva lactuca*	Ulvophyceae	1.00	7.8	86.00	[48]
Cd (II)	*Galdieria sulphuraria*	Cyanidiophyceae	5.00	2.7	24.00	[67]
	*Chlorella* *vulgaris*	Trebouxiophyceae	2.50	7.2	72.00	[42]
	*Coelastrella* sp.	Chlorophyceae	2.50	7.2	82.00	[42]
Pb (II)	*Galdieria sulphuraria*	Cyanidiophyceae	0.10	2.4	0.00	[65]
			5.00	2.7	84.00	[67]
	*Chlorella* *vulgaris*	Trebouxiophyceae	4.83	6.2	98.70	[43]
	*Ulva lactuca*	Ulvophyceae	1.00	7.8	87.00	[48]

## Data Availability

Not applicable.
